# Effect of Rainfall for the Dynamical Transmission Model of the Dengue Disease in Thailand

**DOI:** 10.1155/2017/2541862

**Published:** 2017-08-08

**Authors:** Pratchaya Chanprasopchai, Puntani Pongsumpun, I. Ming Tang

**Affiliations:** ^1^Department of Mathematics, Faculty of Science, King Mongkut's Institute of Technology Ladkrabang, Chalongkrung Road, Ladkrabang, Bangkok 10520, Thailand; ^2^Computational & Applied Science for Smart Innovation Cluster (CLASSIC), Faculty of Science, King Mongkut's University of Technology Thonburi, Bangkok 10140, Thailand

## Abstract

The SEIR (Susceptible-Exposed-Infected-Recovered) model is used to describe the transmission of dengue virus. The main contribution is determining the role of the rainfall in Thailand in the model. The transmission of dengue disease is assumed to depend on the nature of the rainfall in Thailand. We analyze the dynamic transmission of dengue disease. The stability of the solution of the model is analyzed. It is investigated by using the Routh-Hurwitz criteria. We find two equilibrium states: a disease-free state and an endemic equilibrium state. The basic reproductive number (*R*_0_) is obtained, which indicates the stability of each equilibrium state. Numerical results taking into account the rainfall are obtained and they are seen to correspond to the analytical results.

## 1. Introduction

Dengue disease is caused by the dengue virus that is transmitted to human by the bite of a mosquito. The mosquito is the vector of this disease. The spread of dengue disease depends on the contact between the human and the mosquitoes. Therefore, the way to control dengue virus transmission is to either control the mosquito vectors or interrupt the human-vector contact [1]. Outbreaks of dengue disease often occur in most tropical countries around the world, with close to 75% of the global population exposed to the disease living in the Asia-Pacific region [2]. Four serotypes of the dengue virus, DEN1–DEN4, are responsible for the disease in humans. They are all transmitted to human through the bites of infected* Aedes aegypti* and* Aedes albopictus* mosquitoes. When the mosquitoes are in immature or lava stages, they are usually found in water-filled habitats such as water containers close to dwellings of humans. In the adult stages, the mosquitoes may spend most of their lifetimes around the homes of humans. This would lead to the mosquitoes being able to transmit the dengue virus rapidly between the communities.

**Figure 1:**
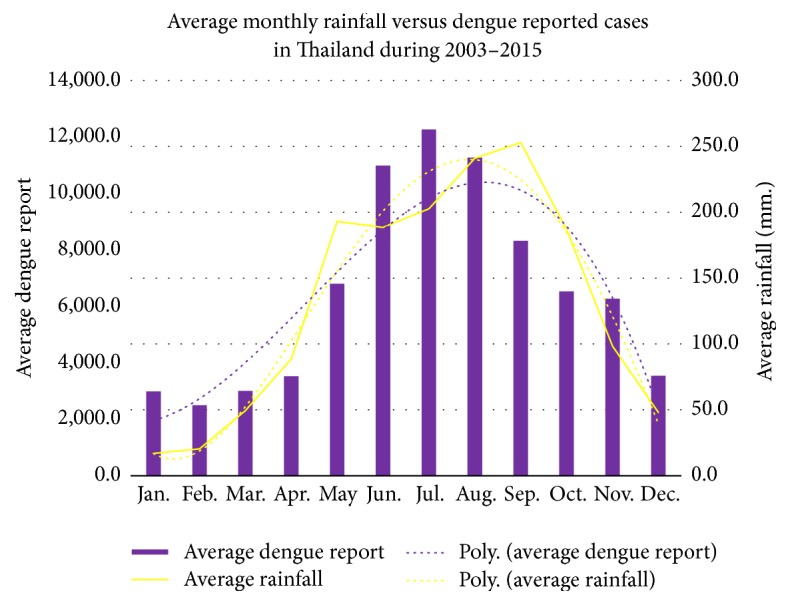
Average monthly rainfall and dengue reported cases during 2003–2015 in Thailand [3].

**Figure 2:**
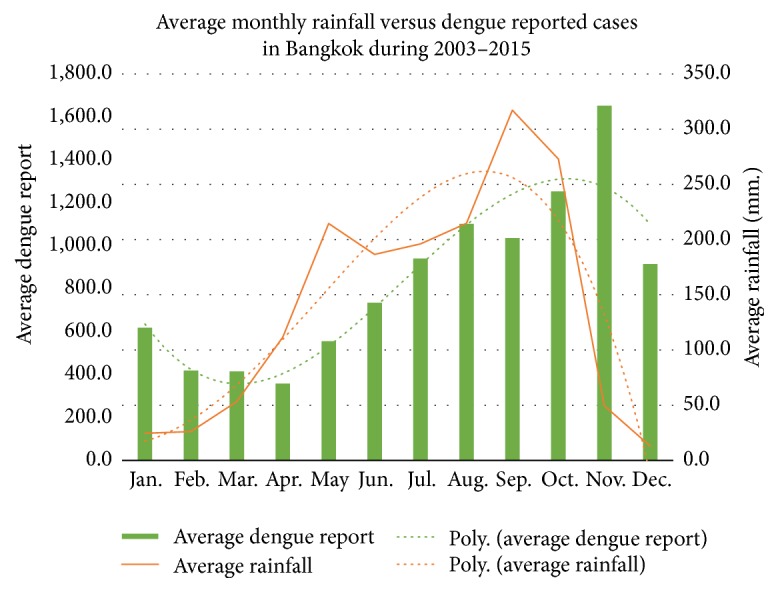
Average monthly rainfall and dengue reported cases during 2003–2015 in Bangkok [3].

In Thailand, dengue disease has been reported nationwide in all parts of Thailand, including the Bangkok metropolitan area in which three forms of dengue disease, dengue fever (DF), dengue hemorrhagic fever (DHF), and dengue shock syndrome (DSS), were reported. The three categories are based on the clinical presentation of patients. The most severe form of dengue disease is DSS [1, 2]. It reappears on a regular basis every year with the peak during the rainy season, June–August. The amount of rainfall is the single most important factor for dengue virus transmission, since this condition is most suitable for mosquitoes to lay their eggs and for the humans and mosquito to come into contact. The historical data in Thailand indicates that the number of reported cases correlates with the average amount of rainfall. The relationships between average monthly dengue reported cases and average monthly amount of rainfall during 2003–2015 in Thailand and Bangkok metropolitan area are presented in Figures 1 and 2, respectively [3].

As we can see from Figures 1 and 2, the correlation between the amount of rainfall and the number of reported cases of dengue disease in the period of this study is cosine function dependence corresponding to the study of Stolwijk et al. [4]. When mosquito bites an infectious human being, the mosquito will be feeding on the infected blood. As a consequence, the mosquito receives the dengue viruses and will be a vector for the transmission of the dengue viruses. The dengue disease epidemic can then be analyzed in order to determine a set of parameter values that will allow a strategy to control the spread of the disease when other factors are taken into account. The mathematical model to be developed will be a SEIR (Susceptible-Exposed-Infected-Recovered) mathematical model. Mathematical models have long been used to describe the dengue transmission.

Esteva and Vargas [5] proposed a model with a constant human population and variable vector population model to describe the transmission of dengue disease and studied the global stability of the endemic equilibrium. Polwiang [6] presented a mathematical model for general vector-host infectious disease and used the reproduction number as a means to evaluate the potential, severity, and persistence of dengue infection. The dengue infection will depend on the seasonal variation of the climate and the rainfall which will affect the breeding pool for the mosquitoes to lay their eggs and to develop into the adult stage. Rodrigues et al. [7] presented disease transmission with the effects of seasonality on the vectorial capacity and, consequently, on the disease development. Using entomological information of the mosquito's behavior under different temperatures and rainfall, the time development of the epidemics was simulated and analyzed. Chompoosri et al. [8] introduced seasonal dengue infection rates in the* Aedes aegypti* mosquitoes to study the dengue infection in suspected patients in 4 central provinces of Thailand. Dengue morbidity rates used for the patients in all 4 provinces were taken to be the highest in rainy season. Kesorn et al. [9] discovered that the* Aedes aegypti* female and larvae mosquito infection rates significantly positively associated with the morbidity rate, where the increasing infection rate of female mosquitoes and larvae led to a higher number of dengue cases. This result supports regarding the largest female populations to be present in the rainy season (May-June) in Thailand, in which the biting activity rate of female mosquitoes increases and more dengue cases occur. Siriyasatien et al. [10] found that female mosquitoes and seasons were strongly correlated with dengue cases in Thailand in which infected female mosquitos together with season are directly correlated to the number of dengue cases. Pongsumpun and Tang [11] analyzed a model when a seasonal variation in the incubation period of the virus while it was developing in the mosquito was included in the model. The annual variation in the length of the extrinsic incubation period was considered by using standard dynamical modeling method to analyze the Susceptible-Exposed-Infected-Recovered (SEIR) model. Chanprasopchai and Pongsumpun [12] used a mathematical model for transmission dynamics of dengue based on the Susceptible-Infected-Recovered (SIR) model. The standard dynamical modeling techniques were used to analyze that model. Relations between each individual variable in the model and the biting rate of mosquito were obtained. Sungchasit et al. [13] later proposed a transmission model of dengue virus in which there were two mosquito species,* Aedes aegypti* and* Aedes albopictus*, causing the infection. Separate SIR (Susceptible-Infected-Recovered) models were proposed to describe the dengue virus transmission by two mosquito species. Pongsumpun and Tang [14] proposed the transmission of dengue hemorrhagic fever by Susceptible-Infected-Recovered model considering the influence of age structure in human population. Human population was divided into two groups, adult and juvenile groups, in order to analyze the dengue disease transmission and the equilibrium state, stability, and numerical calculation were presented. Adams et al. [15] proposed the epidemic pattern observed in Bangkok regarding the result of cross-protective immunity and presented significantly altered changes in the interserotypic immune reaction. They used records of the annual number of confirmed cases of dengue in Bangkok between 1977 and 2000 and used a mathematical model based on standard SIR formulation forced with an annually periodic transmission rate cosine function representing seasonal fluctuations in the vector population.

In this study, we consider the transmission of dengue disease by using mathematical model to investigate the dengue disease mechanism with the effect of rainy season taken into account. The transmission rates of dengue virus vary during the season. The Routh-Hurwitz criteria are applied to analyze the system stability of the SEIR model and the dynamical transmission model of dengue disease is proposed. The equilibrium state and stability, numerical simulation and results, and conclusion are presented.

## 2. Materials and Methods

### 2.1. Mathematical Model

**Figure 3:**
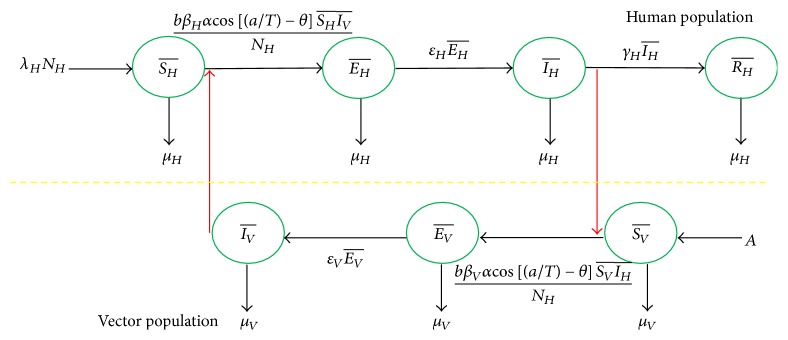
The dynamical transmission of dengue disease.

The SEIR mathematical model consists of two population compartments, human's population and mosquito's population. Human's population includes four epidemiological states, Susceptible human ($\overset{-}{S_{H}}$), Exposed human ($\overset{-}{E_{H}}$), Infected human ($\overset{-}{I_{H}}$), and Recovered human ($\overset{-}{R_{H}}$), whereas mosquito's population is divided into 3 epidemiological states, Susceptible vector ($\overset{-}{S_{V}}$), Exposed vector ($\overset{-}{E_{V}}$), and Infected vector ($\overset{-}{I_{V}}$). The mosquito's population cannot recover from infection which has no recovery epidemiological states. The dynamical transmission between human's population and mosquito's populations is shown in Figure 3.

In the figure, $\overset{-}{S_{H}}\left(t\right)$ is number of susceptible humans at any time *t*, $\overset{-}{S_{V}}\left(t\right)$ is number of susceptible vectors at any time *t*, $\overset{-}{E_{H}}\left(t\right)$ is number of exposed humans at any time *t*, $\overset{-}{E_{V}}\left(t\right)$ is number of exposed vectors at any time *t*, $\overset{-}{I_{H}}\left(t\right)$ is number of infected humans at any time *t*, $\overset{-}{I_{V}}\left(t\right)$ is number of infected vectors at any time *t*, $\overset{-}{R_{H}}\left(t\right)$ is number of recovered humans at any time *t*, *β*_*H*_, *β*_*V*_ are transmission probabilities of dengue virus from vector to human and from human to vector, *ε*_*H*_ is intrinsic incubation rate, *ε*_*V*_ is extrinsic incubation rate, *μ*_*H*_, *μ*_*V*_ are death rates of human and vector, *λ*_*H*_, *γ*_*H*_ are birth rate of human and recovery rate of human, respectively, *A*, *b* are constant recruitment rate and biting rate, respectively, *α*, *θ* are amplitude and horizontal shift of the cosine function, and *a*, *T* are time period and number of time periods.

To simplify the model, we assume that both the human and mosquito populations are constant and there is no vertical transmission; that is, the eggs cannot be infected by sexual contacts between the male and female mosquitoes. It means that total human population is $N_{H}=\overset{-}{S_{H}}+\overset{-}{E_{H}}+\overset{-}{I_{H}}+\overset{-}{R_{H}}$ and total mosquito population is $N_{V}=\overset{-}{S_{V}}+\overset{-}{E_{V}}+\overset{-}{I_{V}}$. The mathematical descriptions of the processes shown in Figure 3 are the seven differential equations given as follows:

(1) dSH¯dt=λHNH−μHSH¯−bβHαcos⁡a/T−θNHSH¯ IV¯,dEH¯dt=bβHαcos⁡a/T−θNHSH¯ IV¯−εHEH¯+μHEH¯,dIH¯dt=εHEH¯−μHIH¯−γHIH¯,dRH¯dt=γHIH¯−μHRH¯,

(2) dIV¯dt=εVEV¯−μVIV¯,dEV¯dt=bβVαcos⁡a/T−θNHSV¯ IH¯−εVEV¯+μVEV¯,dSV¯dt=A−bβVαcos⁡a/T−θNHSV¯ IH¯−μVSV¯.

Since we have assumed that the total human and mosquito populations are constant, we have

(3) dSH¯dt+dEH¯dt+dIH¯dt+dRH¯dt=0,dIV¯dt+dEV¯dt+dSV¯dt=0

with

(4) NV=AμV,λH=μH.

Equations (1) and (2) can be normalized as follows: we first define the normalized variables as

(5) SH=SH¯NH,EH=EH¯NH,IH=IH¯NH,RH=RH¯NH,SV=SV¯NV,EV=EV¯NV,IV=IV¯NV

with

(6) SH+EH+IH+RH=1,SV+EV+IV=1.

Then, there are only five independent variables and only five differential equations are needed. We pick them to be

(7) dSHdt=μH−μHSH−bβHαcos⁡a/T−θNHSHIVNV,dEHdt=bβHαcos⁡a/T−θNHSHIVNV−εHEH−μHEH,dIHdt=εHEH−μHIH−γHIH,dEVdt=bβVαcos⁡aT−θSVIH−εVEV−μVEV,dIVdt=εVEV−μVIV.

We now have five time differential equations involving five independent normalized population groups (*S*_*H*_, *E*_*H*_, *I*_*H*_, *S*_*V*_, *I*_*V*_). We can set the RHS of (7) to zero and find the equilibrium (time-independent) populations. There will be two equilibrium states for each population group, a disease-free equilibrium point and an endemic equilibrium point (one with *I*_*V*_^1(2)^ = 0 and the other ≠ 0). Calling the five equilibrium points for the five populations *X*_*i*_  (*i* = 1,2,…, 5) and letting each of the independent population groups be equal to the equilibrium point plus a perturbation *V*_*i*_ which is time-dependent, we insert these new forms of the solution into (7) and expand the RHS about the equilibrium populations. Doing this, we get the 5 × 5 matrix equation.

(8) $$\begin{array}{c} \frac{dV}{dt}=JV, \end{array}$$

where *J* is the gradient matrix evaluated at the equilibrium points or “the Jacobian matrix.”

### 2.2. Mathematical Analysis for Equilibrium Point

The stability of the solutions of (7) will be considered for 2 cases, one where *θ* ≠ *a*/*T* and one where *θ* = *a*/*T*. It should be noted that when *θ* = *a*/*T*, the argument of the cosine function will be zero, meaning that there are no changes in the rate of infection due to increasing or decreasing rainfall. For *θ* ≠ *a*/*T*, the value of cosine function will change during the rainy season and will vary according to the amount of rainfall, meaning that the rate of transmission will change during the rainy season. The change in rate could be due to the fact that more eggs can be laid or developed quicker or slower depending on the amount of rain that has fallen. It is also well known that climatic factors control the development of the mosquitoes and of the dengue virus.

In either case, the equilibrium points must first be determined. This is done by setting the right-hand side of (7) to zero. Doing this, we obtain two solutions: one will be the disease-free equilibrium point (*E*_1_) and the other will be the endemic equilibrium point (*E*_2_). The two possible solutions depend on whether we take *I*_*V*_ = 0 or *I*_*V*_ ≠ 0. After much work, we find that

(9) E1t=SH1=1,EH1=0,IH1=0,EV1=0,IV1=0,E2t=SH2∗t,EH2∗t,IH2∗t,EV2∗t,IV2∗t,

where

(10) SH2∗t=sec⁡a/T−θεV+μVbαcos⁡a/T−θNHβVεHμH+γH+μHεH+μHμVbαβVεHbαcos⁡a/T−θNVβHεV+NHμHεV+μV,EH2∗t=−seca/T−θμH−2b2α2cos⁡a/T−θ2NVβHβVεHεV+2γH+μHεH+μHμVεV+μV2bαβVεHεH+μHbαcos⁡a/T−θNVβHεV+NHμHεV+μV,IH2∗t=−sec⁡a/T−θμH−2b2α2cos⁡a/T−θ2NVβHβVεHεV+2γH+μHεH+μHμVεV+μV2bαβVγH+μHεH+μHbαcos⁡a/T−θNVβHεV+NHμHεV+μV,EV2∗t=−sec⁡a/T−θNHμHμV−2b2α2cos⁡a/T−θ2NVβHβVεHεV+2γH+μHεH+μHμVεV+μV2bαNVβHεVεV+μVbαcos⁡a/T−θNHβVεHμH+γH+μHεH+μHμV,IV2∗t=−sec⁡a/T−θNHμH−2b2α2cos⁡a/T−θ2NVβHβVεHεV+2γH+μHεH+μHμVεV+μV2bαNVβHεV+μVbαcos⁡a/T−θNHβVεHμH+γH+μHεH+μHμV.

All parameters in the system should be positive definite and the epidemic region of system will be restricted to the region of interest given by

(11) Ω=E1,E2:0≤SH1,SH2∗,EH1,EH2∗,IH1,IH2∗,EV1,EV2∗,IV1,IV2∗≤1.

### 2.3. Mathematical Analysis for Local Stability

The equilibrium states are locally asymptotically stable if all the eigenvalues obtained by solving the eigenvalues equation Det | *J* − *λI* | = 0 have negative imaginary parts. This will be true if the characteristic equation has coefficients which satisfy the Routh-Hurwitz criteria. Performing the calculations, we find that the Jacobian matrix for (7) is just

(12) Jt=−μH−bβHαcos⁡a/T−θNHIVNV000−bβHαcos⁡a/T−θNHSHNVbβHαcos⁡a/T−θNHIVNV−εH+μH00bβHαcos⁡a/T−θNHSHNV0εH−μH+rH0000bβVαcos⁡aT−θ1−IV−EV−εV+μV0000εV−μV.

The stability is usually expressed in terms of what is known as the basic reproduction number. This is the number of secondary infections which is produced by a case of an infection in a population of its infectious period (√*R*_0_). This number is the best indicator of the potential for disease transmission.

**Proposition 1 .**

The equilibrium state *E*_1_ is asymptotically stable when *R*_0_ is lower than 1; that is, *R*_0_ < 1 and *θ* ≠ *a*/*T*.

**Proof**

The local stability of *E*_1_ is governed by linearization of (7). Rearranging the expressions for the equilibrium values of *I*_*H*_ or *E*_*H*_ so that they would be in a form *∝R* − 1, we find that *R*_0_ will be of the following form:

(13) $$\begin{array}{c} R_{0}=\frac{b^{2}\alpha^{2}\cos{\left[2\pi t/T-\theta \right]}^{2}N_{V}\beta_{H}\beta_{V}\varepsilon_{H}\varepsilon_{V}}{N_{H}\mu_{V}\left(\gamma_{H}+\mu_{H}\right)\left(\varepsilon_{H}+\mu_{H}\right)\left(\varepsilon_{V}+\mu_{V}\right)}. \end{array}$$

The eigenvalues of (12) are used to evaluate the disease-free equilibrium point which is determined by solving

(14) $$\begin{array}{c} Det\left|\;J-\lambda I\;\right|=0, \end{array}$$

where  *J*_*E*_1__ is the Jacobian matrix at the equilibrium point *E*_1_, *λ* are eigenvalues, and *I*_5_ is identity 5 × 5 matrix.

Evaluating the determinant of (12), we obtain the following characteristic equation:

(15) $$\begin{array}{c} \left(\;\lambda +\mu_{H}\;\right)\left(\;\lambda^{4}+e_{1}\lambda^{3}+e_{2}\lambda^{2}+e_{3}\lambda^{1}+e_{4}\;\right)=0, \end{array}$$

where

(16) e1=γH+εH+εV+2μH+μV,e2=εHγH+εV+μH+2μV+εVγH+2μH+μV+γHμH+μH2+2γHμV+4μHμV+μV2,e3=εHγHεV+εVμH+2γHμV+εVμV+2μHμV+μV2+εVγHμH+μH2+γHμV+2μHμV+μHμVμH+μV+γH2μHμV+μV2,e4=b2α2cos⁡2πtT−θ2NVβHβVεHεV+γH+μHεH+μHμVεV+μV.

Routh-Hurwitz criteria required for all of the eigenvalues (solutions) defined by (12) are negative real parts and the coefficients must satisfy all conditions given as follows:

(17) e1>0,e3>0,e4>0,e1e2e3>e32+e12e4.

**Figure 4:**
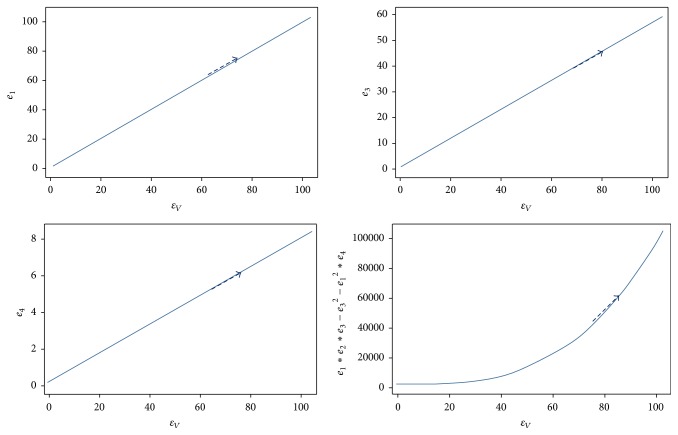
All parameters spaces of disease-free equilibrium of *E*_1_ satisfy the Routh-Hurwitz criteria. All parameter values are *N*_*H*_ = 9,200, *b* = 1/3, *μ*_*H*_ = 1/(70*∗*365), *γ*_*H*_ = 1/3, *β*_*H*_ = 0.1, *β*_*V*_ = 0.1, *μ*_*V*_ = 1/14, *ε*_*V*_ = 0.1428, *ε*_*H*_ = 0.1667, and *A* = 500.

When this happens and for *R*_0_ < 1, disease-free equilibrium will be stable as is seen in Figure 4.

**Proposition 2 .**

The equilibrium state *E*_2_ is asymptotically stable when *R*_0_ is higher than 1; *R*_0_ > 1 and *θ* ≠ *a*/*T*.

**Proof**

The local stability of *E*_2_ is governed by linearization of (7). The characteristic equation is now

(18) $$\begin{array}{c} \left(\;\lambda^{5}+e_{1}\lambda^{4}+e_{2}\lambda^{3}+e_{3}\lambda^{2}+e_{4}\lambda^{1}+e_{5}\;\right)=0, \end{array}$$

where

(19) e1=−η1η2η3η4η5+η1η4η5η9η3−μVη8+η3η8η4η5+η4η7η1η3η4η5,e2=−η1η2η4η5η9η3−μVη8+η3η8η4η5+η4η7+η1−η9μVη8η4η5+η3η8η4η7η9η3−μVη8+η3η8η4η5+η4η7η1η3η4η5,e3=η1η8η4η7η9η3−μVη8−η9μVη8η3η8η4η5+η4η7−η1η2−η9μVη8η4η5+η3η8η4η7+η9η3−μVη8+η3η8η4η5+η4η7η1η3η4η5,e4=−η1η9μVη82η4η7+η1η82η4η5η6−η1η2η8η4η7η9η3−μVη8−η9μVη8η8η4η5+η4η7η1η3η4η5,e5=NHμHγH+μHεH+μHμVb2α2cos⁡2πt/T−θ2NVβHβVεHεV−γH+μHεH+μHμVεV+μVbαcos⁡2πt/T−θNHβVεHμH+γH+μHεH+μHμV,

where

(20) η1=NVβHβV2εH2εV,η2=−γH−μH,η3=−εV−μV,η4=bαcos⁡2πtT−θNVβHεV+NHεVμH+NHμHμV2bαcos⁡2πtT−θNHβVεHμH+γHεHμV+γHμHμV+εHμHμV+μH2μV2,η5=bαcos⁡2πtT−θNHβVεHεVμH+γHεHεVμV+bαcos⁡2πtT−θNHβVεHμHμV+γHεVμHμV+εHεVμHμV+εVμH2μV+γHεHμV2+γHμHμV2+εHμHμV2+μH2μV2,η6=bαcos⁡2πtT−θNVβHγHεHεVμV+bαcos⁡2πtT−θNVβHγHεVμHμV+bαcos⁡2πtT−θNVβHεHεVμHμV+NHγHεHεVμHμV+bαcos⁡2πtT−θNVβHεVμH2μV+NHγHεVμH2μVNHεHεVμH2μV+NHεVμH3μV+NHγHεHμHμV2+NHγHμH2μV2+NHεHμH2μV2+NHμH3μV2,η7=b2α2cos⁡2πtT−θ2NHNVβHβVεHεVμH+bαcos⁡2πtT−θNHβVεHεVμH2+γHεHεVμHμV−NHγHεHεVμHμV+bαcos⁡2πtT−θNHβVεHμH2μV+γHεVμH2μV−NHγHεVμH2μV+εHεVμH2μV−NHεHεVμH2μV+εVμH3μV−NHεVμH3μV+γHεHμHμV2−NHγHεHμHμV2+γHμH2μV2−NHγHμH2μV2+εHμH2μV2−NHεHμH2μV2+μH3μV2−NHμH3μV2,η8=εV+μV,η9=εH+μH.

The endemic equilibrium point of local stability of the system will have negative real parts when the coefficients in the characteristic equation (18) satisfy the Routh-Hurwitz conditions now given by

(21) e1>0,e2>0,e3>0,e4>0,e5>0,e1e2e3−e32−e12e4>0,e1e4−e5e1e2e3−e32−e12e4−e5e1e2−e32−e1e52>0.

**Figure 5:**
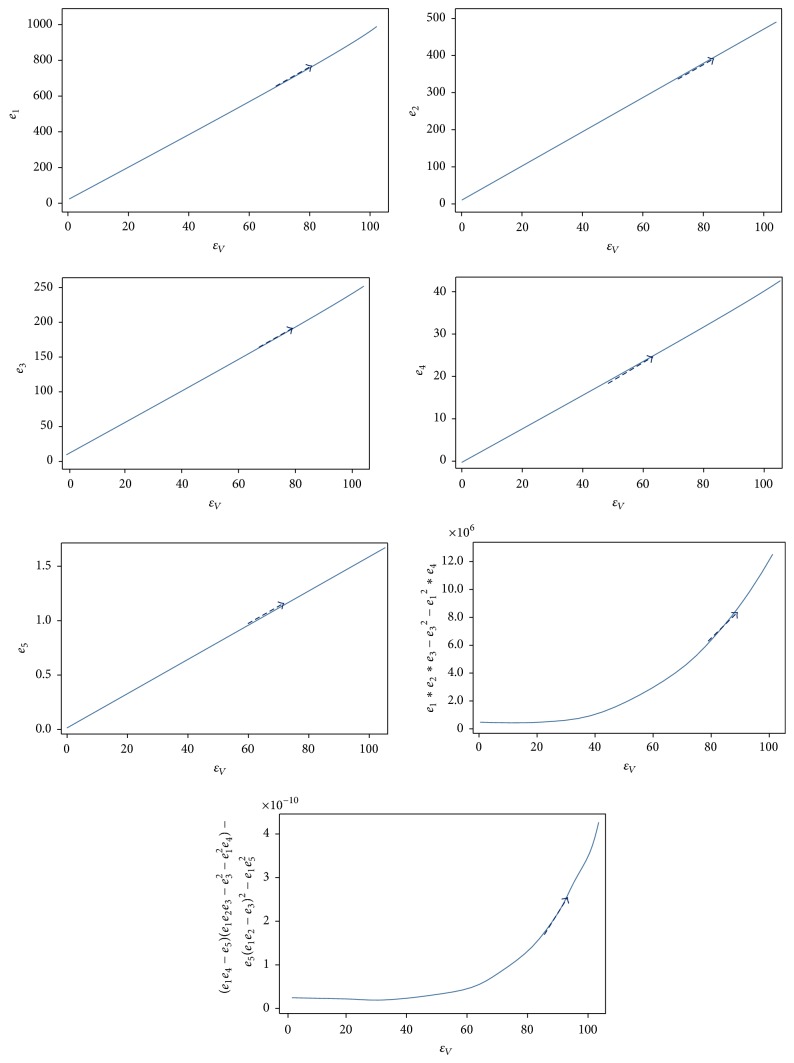
All parameters spaces of endemic equilibrium of *E*_2_ satisfy the Routh-Hurwitz criteria. All parameter values are *N*_*H*_ = 9,200, *b* = 1/3, *μ*_*H*_=1/(70*∗*365), *γ*_*H*_ = 1/3, *β*_*H*_ = 0.5, *β*_*V*_ = 0.3, *μ*_*V*_ = 1/14, *ε*_*V*_ = 0.1428, *ε*_*H*_ = 0.1667, and *A* = 500.

All conditions of (21) are satisfied for endemic equilibrium point as is evident by the behaviors seen in Figure 5. Next, we consider the case of *θ* = *a*/*T*; (7) will be the standard case of SEIR model, where no effects of rainfall are taken into account. As a result, the mathematical equations describing the model are

(22) dSHdt=μH−μHSH−bβHNHSHIVNV,dEHdt=bβHNHSHIVNV−εHEH−μHEH,dIHdt=εHEH−μHIH−γHIH,dEVdt=bβVSVIH−εVEV−μVEV,dIVdt=εVEV−μVIV.

*R*_0_ obtained from (22) is as follows:

(23) $$\begin{array}{c} R_{0}=\frac{b^{2}N_{V}\beta_{H}\beta_{V}\varepsilon_{H}\varepsilon_{V}}{N_{H}\mu_{V}\left(\gamma_{H}+\mu_{H}\right)\left(\varepsilon_{H}+\mu_{H}\right)\left(\varepsilon_{V}+\mu_{V}\right)}. \end{array}$$

## 3. Numerical Results

The transmission of dengue disease in this study is based on the SEIR model. The susceptible class will be people who have no immunity and who are not infectious. Human beings infected are people who are infectious, that is, able to pass on the virus onto the mosquitoes. The infectious period will be taken to be the period during which the person appears to be sick, a period of one to two weeks. When human gets well, the patient passes into the recovery class with lifelong immunity to the virus. In this study, the numerical simulations assume the following values of the parameters; *μ*_*H*_ = 1/(70*∗*365) per day corresponding to a life expectancy of 70 years in Thai people, *A* = 5,000 corresponding to constant recruitment rate, *N*_*H*_ = 92,000 corresponding to total number of human population, and *b* = 1/3 corresponding to biting rate of vector population.

**Figure 6:**
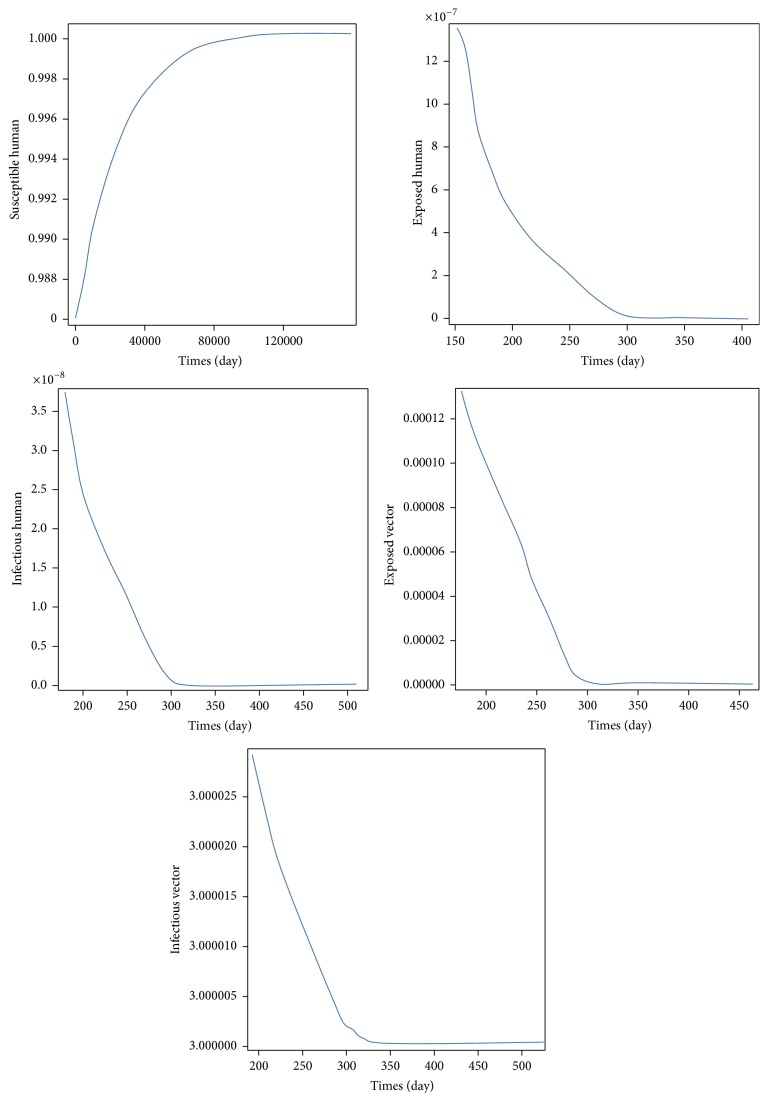
The trajectories of the time evolutions of the five population groups, *S*_*H*_, *E*_*H*_, *I*_*H*_, *E*_*V*_, and *I*_*V*_, towards the disease-free equilibrium.

**Figure 7:**
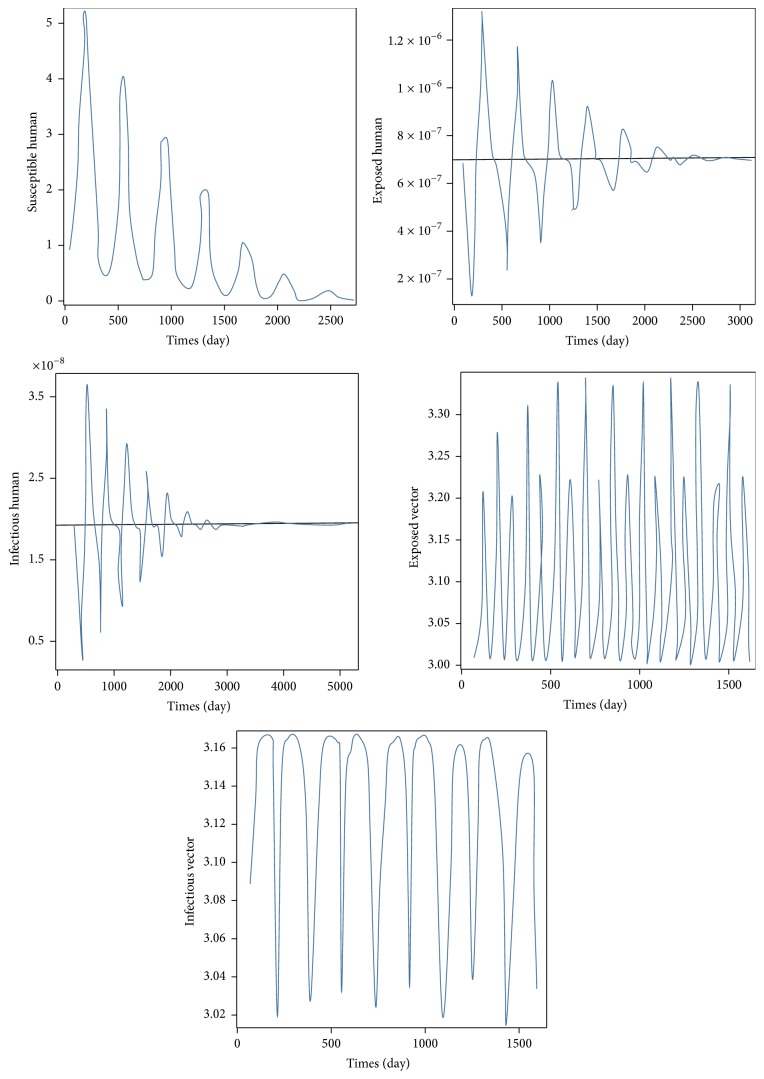
The trajectories of the time evolution of *S*_*H*_, *E*_*H*_, *I*_*H*_, *E*_*V*_, and *I*_*V*_ based on numerical solving.

**Figure 8:**
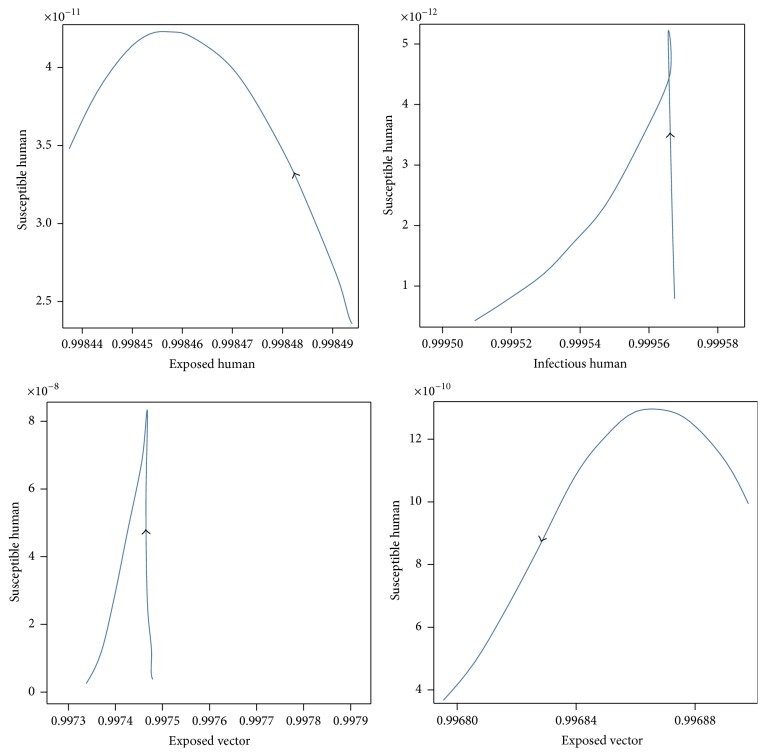
The trajectories of the numerical solutions of dengue disease for disease-free equilibrium projected onto (*S*_*H*_, *E*_*H*_), (*S*_*H*_, *I*_*H*_), (*S*_*H*_, *E*_*V*_), and (*S*_*H*_, *I*_*V*_).

**Figure 9:**
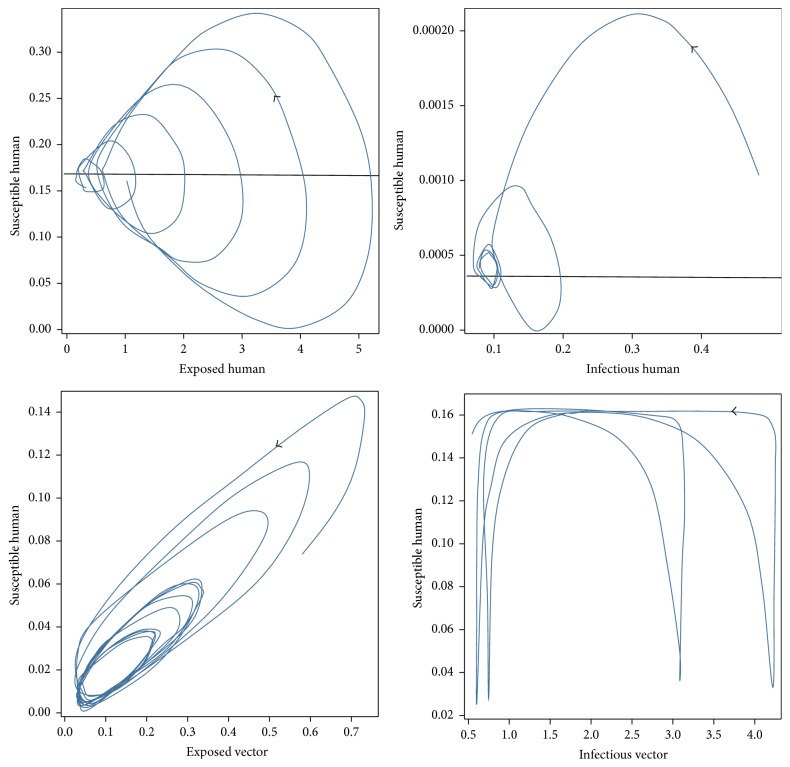
The trajectories of the numerical solutions of dengue disease for endemic equilibrium projected onto (*S*_*H*_, *E*_*H*_), (*S*_*H*_, *I*_*H*_), (*S*_*H*_, *E*_*V*_), and (*S*_*H*_, *I*_*V*_).

**Figure 10:**
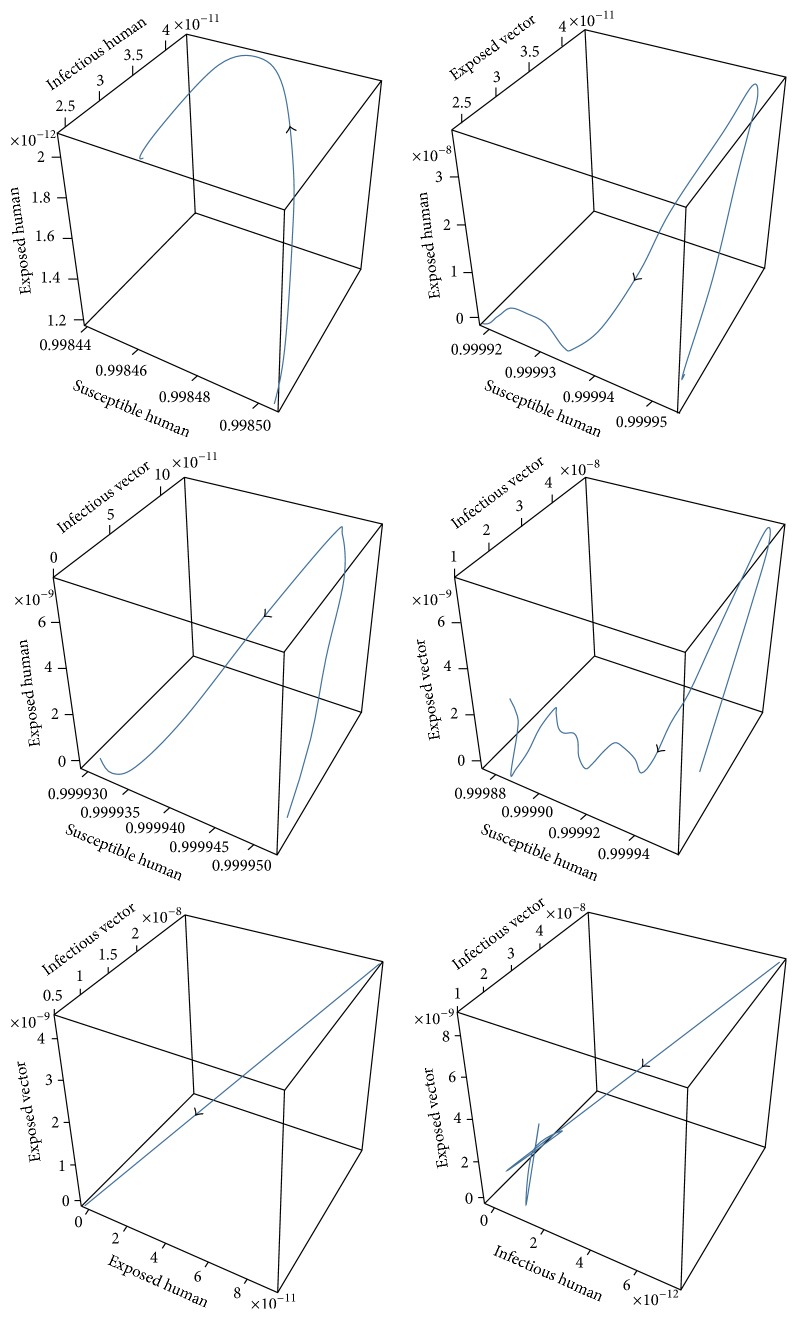
The trajectories of the numerical solutions of dengue disease for disease-free equilibrium projected onto (*S*_*H*_, *E*_*H*_, *I*_*H*_), (*S*_*H*_, *E*_*H*_, *E*_*V*_), (*S*_*H*_, *E*_*H*_, *I*_*V*_), (*S*_*H*_, *E*_*V*_, *I*_*V*_), (*E*_*H*_, *E*_*V*_, *I*_*V*_), and (*I*_*H*_, *E*_*V*_, *I*_*V*_).

**Figure 11:**
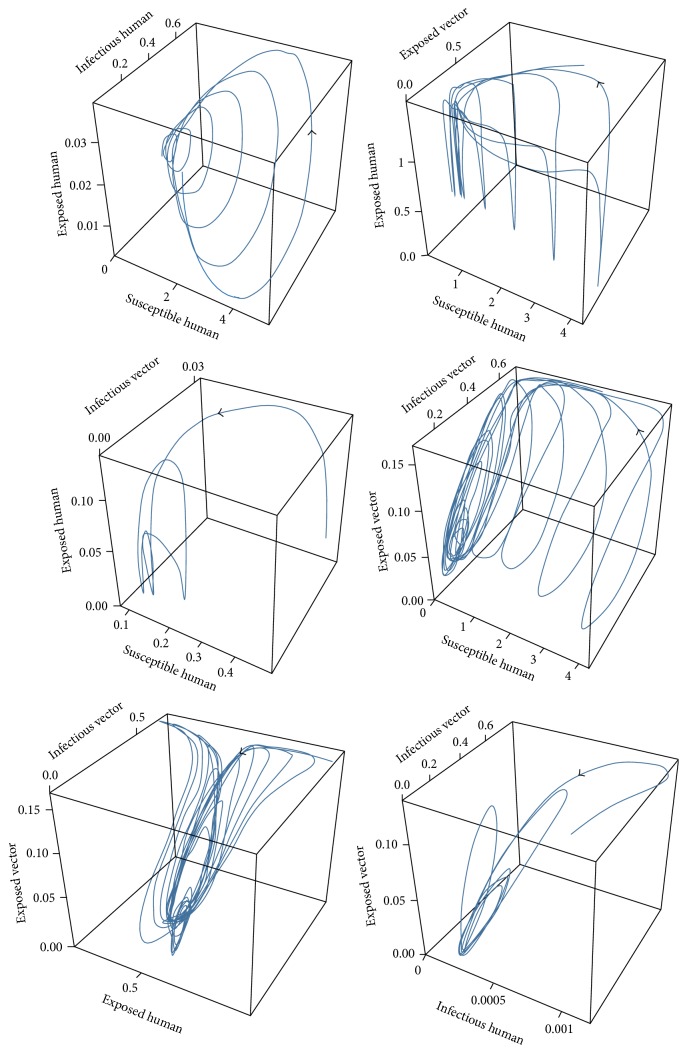
The trajectories of the numerical solutions of dengue disease for endemic equilibrium projected onto (*S*_*H*_, *E*_*H*_, *I*_*H*_), (*S*_*H*_, *E*_*H*_, *E*_*V*_), (*S*_*H*_, *E*_*H*_, *I*_*V*_), (*S*_*H*_, *E*_*V*_, *I*_*V*_), (*E*_*H*_, *E*_*V*_, *I*_*V*_), and (*I*_*H*_, *E*_*V*_, *I*_*V*_).

For case 1, the values of the parameters of case 1 for disease-free equilibrium are *μ*_*V*_ = 1/14, *γ*_*H*_ = 1/3, *β*_*H*_ = 0.1, *β*_*V*_ = 0.1, *ε*_*V*_ = 0.1428, and *ε*_*H*_ = 0.1667 which will lead to *R*_0_ < 1, while the set of values of the parameters of case 2 are *μ*_*V*_ = 1/14, *γ*_*H*_ = 1/3, *β*_*H*_ = 0.5, *β*_*V*_ = 0.3, *ε*_*V*_ = 0.1428, and *ε*_*H*_ = 0.1667 which will lead to *R*_0_ > 1. The trajectories of the numerical solutions for case 1 and for case 2 of *S*_*H*_, *E*_*H*_, *I*_*H*_, *E*_*V*_, and *I*_*V*_ are shown in the Figures 6 and 7, respectively. The trajectories of the numerical solutions for case 1 and case 2 plotted in the 2D (*S*_*H*_, *E*_*H*_), (*S*_*H*_, *I*_*H*_), (*S*_*H*_, *E*_*V*_), and (*S*_*H*_, *I*_*V*_) planes are shown in Figures 8 and 9, respectively. The trajectories of the numerical solutions for case 1 and case 2 in the 3D (*S*_*H*_, *E*_*H*_, *I*_*H*_), (*S*_*H*_, *E*_*H*_, *E*_*V*_), (*S*_*H*_, *E*_*H*_, *I*_*V*_), (*S*_*H*_, *E*_*V*_, *I*_*V*_), (*E*_*H*_, *E*_*V*_, *I*_*V*_), and (*I*_*H*_, *E*_*V*_, *I*_*V*_) spaces are shown in Figures 10 and 11, respectively.

## 4. Discussion and Conclusion

The effect of rainfall on the dynamic transmission of dengue disease in Thailand has been studied using the SEIR model to model the dynamics of the dengue epidemic in Thailand. The analysis is based on using the Routh-Hurwitz criteria to establish the local asymptotic stability of the equilibrium points. Two equilibrium points were found: a disease-free equilibrium point and an endemic equilibrium point. The disease-free equilibrium point, *E*_1_, is locally asymptotically stable for *R*_0_ < 1 and *θ* ≠ *a*/*T*. The set of differential equations for the SEIR model of the dengue infections were solved for different sets of numerical values of the parameters to obtain the different trajectories of the different population groups in the model. The trajectories were projected into the 2D (*S*_*H*_, *E*_*H*_), (*S*_*H*_, *I*_*H*_), (*S*_*H*_, *E*_*V*_), and (*S*_*H*_, *I*_*V*_) planes and onto the 3D (*S*_*H*_, *E*_*H*_, *I*_*H*_), (*S*_*H*_, *E*_*H*_, *E*_*V*_), (*S*_*H*_, *E*_*H*_, *I*_*V*_), (*S*_*H*_, *E*_*V*_, *I*_*V*_), (*E*_*H*_, *E*_*V*_, *I*_*V*_), and (*I*_*H*_, *E*_*V*_, *I*_*V*_) spaces. These trajectories are shown in Figures 4, 6, 8, and 10, respectively. When the values of the parameters are such that *R*_0_ > 1 and *θ* ≠ *a*/*T*, then the trajectories ending at the endemic equilibrium point, *E*_2_, are described in Figures 5, 7, 9, and 11. The numerical results correspond to Propositions 1 and 2.

Looking at the figures, we see that everything is determined by whether *R*_0_ < 1 or *R*_0_ > 1; in the first case, the equilibrium state is the disease-free state, while in the second case, it is the endemic equilibrium state. Everything depends on the form of the expression for *R*_0_. In summary, *R*_0_ in SEIR model which includes the effect of rainfall is given by (24), while *R*_0_ in SEIR model which ignores the effect of rainfall is given by expression (25). *R*_0_ value of the SIR model of Esteva and Vargas [5] is given by (26) and the simplest expression is given by Rodrigues et al. [7] in (27).

(24) R0=b2α2cos⁡2πt/T−θ2NVβHβVεHεVNHμVγH+μHεH+μHεV+μV,

(25) R0=b2NVβHβVεHεVNHμVγH+μHεH+μHεV+μV,

(26) R0=b2βHβVNHA/μVNH+m2μVγH+μH,

(27) R0=b2βHβVNVNHμVγH+μH.

In expression (26), *m* is the number of alternative sources of blood, that is, other animals.

**Figure 12:**
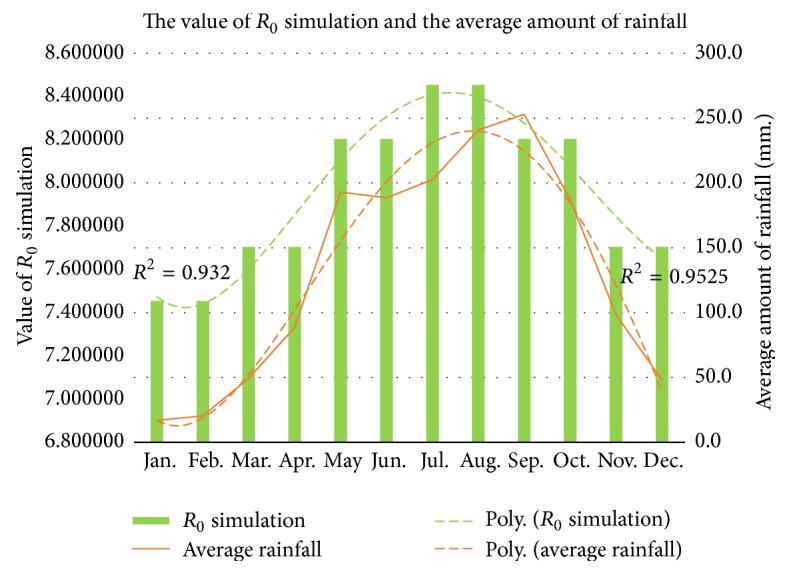
The value of *R*_0_ simulation of endemic equilibrium state and the average amount of rainfall.

In (24), *R*_0_ value is considered the effect of rainfall, where Thailand has correlation between rainfall and the prevalence of clinical cases of dengue [16]. Thailand's historical data indicate that rainfall was associated with dengue in many regions, for example, southern [17], northern [18], northeastern [19], and central [20] regions. Dengue disease fluctuation is related to climate variability and seasonal factor is taken into account [10, 21–25] in which the dengue virus transmission is considered as a cosine function [15, 26]. When *R*_0_ > 1, this will increase the opportunity of the outbreak situation. *R*_0_ is an important indicator, where the realistic controlling of the value of *R*_0_ will improve the way to control the outbreak. The value of *R*_0_ simulation of endemic equilibrium state and the average amount of rainfall are shown in Figure 12. It is indicated that there is a relation between the value of *R*_0_ and the average amount of rainfall.

In addition, the suitable ways to control the dengue disease are environmental management to prevent mosquitoes from laying their eggs and breeding and using of personal household protection to prevent contact between human and mosquito [1, 2]. In the present paper, we did not explicitly take into account the egg and aquatic stages of the mosquito development (see (2)) as was done by Erickson et al. [27] and Moulay et al. [28]. The lack of these classes precludes any discussion of the vertical transmission of the disease, since the “sexual” transmission (evidenced by the presence of the DNA fragments of the dengue virus in the larvae and pupae [29]) occurs at these stages.
